# Neurological manifestations of Varicella zoster infection: A case series

**DOI:** 10.4102/jcmsa.v2i1.76

**Published:** 2024-07-08

**Authors:** Azhar Rylands, Shakeel Hoosain

**Affiliations:** 1 Department of Internal Medicine, Faculty of Health Sciences, University of Cape Town, Cape Town, South Africa

**Keywords:** Varicella Zoster, neurology, transverse myelitis, encephalitis, meningitis, stroke, manifestations, disease

## Abstract

**Introduction:**

Varicella zoster virus (VZV) causes infection of the central nervous system, which manifests as various neurological syndromes. In this case series, we discuss three different clinical presentations of VZV reactivation (herpes zoster). The main clinical feature of herpes zoster is a pruritic vesicular rash, present in a dermatomal distribution, which occurs after reactivation of the virus because of immunosuppression.

**Patients presentation:**

We report on three individual cases of complicated herpes zoster that resulted in various neurological pathologies. The patients described in this case series each received a lumbar puncture with cerebrospinal fluid analysis, which showed a positive varicella zoster polymerase chain reaction test (VZV PCR).

**Management and outcome:**

All three patients received intravenous acyclovir as their core component of treatment. In addition to this, some received other adjunctive treatments, mainly corticosteroids. Two patients achieved disease resolution while one patient demised.

**Conclusion:**

The case series highlights the various ways in which VZV can affect the central nervous system (CNS) such as meningitis, encephalitis, myelitis and arteritis.

**Contribution:**

This is a brief overview of the clinical manifestations of VZV affecting the CNS in patients presenting to a regional hospital within the Western Cape province in South Africa.

## Introduction

Varicella zoster virus (VZV) is a pathogenic double-stranded deoxyribonucleic acid (DNA) virus that belongs to the genus Varicellovirus, subfamily Alphaherpesvirinae and family Herpesviridae.^1^ Varicella zoster virus infection typically includes a primary infection, commonly occurring in childhood, known as varicella or chickenpox, which then becomes latent in the dorsal root, cranial nerve and autonomic ganglia along the neuroaxis. Reactivation of this disease is known as herpes zoster or shingles.^2^ Herpes zoster is a painful vesicular rash, which is usually localised to a specific dermatome of the body.^3^ Herpes zoster infection can be complicated by various neurological manifestations including arteritis, myelitis, meningitis, and encephalitis.^4^ Complicated herpes zoster disease can be present in immunocompetent and immunosuppressed individuals with higher mortality and morbidity rates in immunosuppressed individuals because of decreased T-cell mediated immunity.^5,6^ Cerebrospinal fluid (CSF) analysis and clinical features of neurological deficits are fundamental in diagnosing neurological manifestations of the disease, as well as the presence of abnormal findings on special imaging. Cerebrospinal fluid of affected individuals commonly demonstrate a lymphocytic predominant pleocytosis, a raised protein and a detection of VZV DNA through polymerase chain reaction tests (PCR).^4^ Clinical features of neurological deficits include fever, vomiting, headaches, new-onset seizures, cranial nerve palsies, strokes and altered mental states.^7^ The treatment of choice for VZV encephalitis and its associated neurological complications is intravenous acyclovir, with longer courses being used for immunocompromised individuals. Corticosteroids have been used as an adjunctive treatment for associated inflammation.^7^ The aim of this case series is to describe the various neurological manifestations of herpes zoster infection. Its further aim is for health professionals to gain understanding and knowledge of this disease as well as gaining the ability to better diagnose and approach treatment of the disease within the population.

## Case presentations

### Case 1

A 29-year-old female, with a history of previous neurological insult 3 years prior, labelled as a ‘stroke’ and residual facial weakness, presented to the emergency unit (EU) at New Somerset Hospital with a 2-week history of new-onset bilateral lower limb weakness with paraesthesia, urinary incontinence, dizziness, vertigo and blurred vision, which was preceded by 1 week of viral upper respiratory symptoms and fever. The patient initially presented to a local day hospital where she was given prednisone, amoxicillin and paracetamol with no resolution of symptoms.

Upon arrival at the EU triage, the patient was recorded to have a heart rate of 103 beats per minute (bpm), a respiratory rate of 16 breaths per minute, a blood pressure (BP) of 116/70 mmHg, a temperature of 36.5 °C, pulse oximetry of 99% on room air and was recorded to be fully alert. Random blood glucose was not documented. Further side room investigations included a urine dipstick test, which was normal and a negative rapid pregnancy test. Additionally, the patient was tested for human immunodeficiency virus (HIV) using an HIV Tri-Line rapid test device. The result was positive. The patient reported she had not previously been aware of her status and reported not being on antiretroviral treatment (ART).

General examination findings included a vesicular rash on the T4–T5 dermatome on the chest wall and back of the patient. Neurological examination findings recorded at the time of presentation included: Glasgow Coma Scale of 15/15 and a left cranial nerve 7 fall-out. Upper limb hypotonia was present on the right side with normal left upper limb tone. Lower limb spasticity was present with decreased power in lower limbs bilaterally, with a grading of 3/5 in the right leg and 2/5 in the left leg. Normal upper and lower limb reflexes were present. Cerebellar signs were present in the form of upper and lower limb dysdiadochokinesia, down-beat nystagmus, intention tremor, finger-nose and heel-shin ataxia, with more prominent signs on the right side. Back pain at the location of the T5 dermatome was reported with an associated clinical sensory level. Cardiovascular, respiratory and abdominal examinations were documented as unremarkable. The patient was admitted to the Department of Internal Medicine where further investigations were performed.

Initial blood tests showed a microcytic anaemia with a low haemoglobin level of 11.6 g/dL and a low mean corpuscle volume of 78.4 femtoliters (fL). Furthermore, there were no abnormalities of white cells or platelet counts. Renal function was recorded as normal. A low cluster of differentiation 4 (CD4) count of 186 was documented indicating advanced staged HIV. The serum cryptococcal antigen, syphilis serology and viral hepatitis tests were negative. Varicella zoster virus immunoglobulin G (IgG), however, tested positive in the serum. A pre- and post-contrast computed tomography (CT) brain scan was done, which showed an area of low density in the superomedial right cerebellar hemisphere.

This was then followed up with a lumbar puncture that showed the following results: Normal CSF protein of 0.37 g/L, polymorphs of 0 and lymphocytes of 1 µL. CSF Cryptococcal antigen, tuberculosis (TB) GeneXpert and Treponemal antibodies were negative. Varicella zoster polymerase chain reaction test and Polyomavirus JC Virus PCR-tested positive in the CSF. A diagnosis of herpes zoster encephalitis complicated by cerebellar infarct and transverse myelitis was made.

The patient was initiated on intravenous (IV) acyclovir for 14 days, oral prednisone and received inpatient rehabilitation by the physiotherapy and occupational therapy teams. Her lower limb weakness resolved as her admission progressed and she was able to walk independently by the time of her discharge. Residual lower limb weakness was documented at the time of discharge with a power grading of 5/5 in the right leg and 4/5 in the left leg.

### Case 2

Twenty three-year-old immunocompetent male presented to the EU at New Somerset Hospital with a 1-day history of acute onset confusion and bizarre and aggressive behaviour. The patient was brought in by his family with reports of abrupt change in behaviour with the presence of auditory and visual hallucinations. The patient was combative with the EU staff and required chemical sedation. He was treated in a private health facility 1 week prior for sinusitis and otitis media. He was given co-amoxiclav and prednisone at the time.

Upon presenting to the EU triage, he had a heart rate of 75 bpm, a respiratory rate of 26 breaths per minute, a BP of 150/96 mmHg, a temperature of 36.6 °C and pulse oximetry of 99% on the room air. Random blood glucose was 5.3 mmol/L. Urine dipstick was clear, and urine toxicology was negative for the common illicit and high schedule drugs. He was tested for HIV using the HIV Tri-Line rapid test device in the EU and was documented as negative. The patient was admitted to the Department of Internal Medicine for further investigations and treatment.

Upon general examination, a vesicular rash was observed on the T10 dermatome on the patient’s back. The patient was confused and disorientated; however, he managed to engage and follow with basic instructions. No obvious cranial nerve abnormalities were noted. Tone, power and reflexes globally were examined as normal. Sensation noted to be intact with no proprioceptive loss or co-ordination abnormalities. Respiratory, cardiovascular and abdominal examinations were unremarkable.

A set of blood tests were performed to investigate for the cause of the delirium.

Biochemistry showed a normal renal function, a normal C-reactive protein (CRP), normal thyroid function, normal liver enzymes, a raised white cell count of 15.1 × 10^9^ with a normal haemoglobin level and platelet count. Furthermore, an HIV antibody/antigen enzyme-linked immunosorbent (ELISA) test was performed, which was negative. A non-contrast CT brain scan was performed, which showed no obvious structural intracranial pathology.

The lumbar puncture showed a high protein count of 1.01 g/L with a lymphocytic pleocytosis of 499 cells/µL, findings consistent with a viral infection. Cryptococcal antigen, TB GeneXpert, PCR bacterial meningitis panel and Treponemal antibodies were tested on the CSF and were negative. Viral panel testing was performed on the CSF specimen, which included Herpes Simplex 1&2 PCR, Cytomegalovirus, Epstein-Barr virus and Enterovirus. All tested negative except for the VZV PCR, which was positive. A diagnosis of herpes zoster encephalitis complicated by acute psychosis was made.

The patient was initiated on a 10-day course of IV acyclovir. The patient’s delirium resolved as the treatment progressed. On day 5 of treatment, the lumbar puncture was repeated, and a decrease in the CSF protein was noted. There was a reduction from the 1.01 g/L of protein on the day of admission, to 0.49 g/L on day 5 of treatment. He was discharged once 10 days of the IV acyclovir was completed, having regained his full mental state with no complications.

### Case 3

A 35-year-old female presented to the EU at New Somerset Hospital with a 5-day history of right ear pain and hearing loss with associated mucopurulent discharge, a sore throat and hoarseness and a vesicular painful rash on the right side of her face. She was initially seen at a local day hospital and was given an adrenaline nebuliser, prednisone, ceftriaxone, diclofenac and paracetamol before transfer to New Somerset Hospital.

Upon arrival at the EU triage, her vital signs included a heart rate of bpm, a respiratory rate of 18 breaths per minute, BP of 165/101 mmHg, a temperature of 38.2 °C, pulse oximetry of 96% on room air and a random blood glucose of 7.8 mmol/L. Rapid urine dipstick and pregnancy test were negative, and HIV was found to be positive after the use of an HIV Tri-Line rapid test device. She reported not knowing her HIV status prior and reported not being on ART. An electrocardiogram (ECG) was performed, which showed a sinus tachycardia.

**TABLE 1:** Clinical data of the three selected patients in the case series.

Case	Gender	Age	Immune status	Vesicular dermatomal rash	Length of admission (days)	Main clinical presentation	Core treatment	Outcome
1	Female	29	HIV-positive	Yes	16	Bilateral lower limb paresis, urinary incontinence, dizziness, blurred vision	14 days of Acyclovir and steroids	Improved with mild residual weakness
2	Male	23	HIV-negative	Yes	11	Acute psychosis	10 days of Acyclovir	Resolved
3	Female	35	HIV-positive	Yes	18	Herpes Zoster Oticus, vomiting, headache, sore throat	Acyclovir, Ceftriaxone and steroids	Demised

General examination upon first presentation included findings of cervical lymphadenopathy, oral candida and a vesicular rash with a right lower motor neuron facial palsy and a deficit in eyelid closure. An ear, nose and throat (ENT) examination included findings of an exudative tonsillitis and an 80% stenosis of the right external ear canal with oedema and multiple vesicles. The tympanic membrane was poorly visualised. Cardiovascular, respiratory and abdominal examinations were unremarkable. A diagnosis of Ramsay-Hunt syndrome was made when the patient was admitted to the Department of Internal Medicine for admission and further management. Acyclovir IV and ceftriaxone to cover for a secondary bacterial infection was initiated as treatment.

Biochemistry on admission showed normal renal function, a raised CRP of 181 mg/L, a normal full blood count, a low CD4 count of 112, a negative cryptococcal antigen test and a positive HIV antibody/antigen ELISA test. An initial pre- and post-contrast CT brain scan was performed, which showed no features of intracranial pathology or mass effect.

The lumbar puncture showed a raised CSF protein of 2.98 g/L with a lymphocytic predominant pleocytosis, which included raised lymphocytes of 431 cells/µL and raised polymorphs of 217 cells/µL. A CSF adenosine deaminase was performed, which showed a normal result of 2.5 µL. Cerebrospinal fluid Treponemal antibodies, bacterial meningitis PCR panel, TB GeneXpert and Herpes Simplex PCR were negative. Varicella zoster polymerase chain reaction test was positive. Varicella zoster virus encephalitis with Ramsay-Hunt syndrome was confirmed.

Given the CSF profile, a differential diagnosis of tuberculosis meningitis was considered prior to the full result panel becoming available. This was excluded with a negative urinary mycobacterial lipoarabinomannan test, a negative chest X-ray and negative sputum PCR test.

On day 4 of admission, after review by the ENT team, oral prednisone, ciprofloxacin ear drops, chlorohexidine topical ear ointment and artificial eye drops were initiated and instruction for regular gentle ear cleaning and eye care was given.

Unfortunately, her hospital admission was complicated by sudden episodes of seizures with a raised BP. On day 7 of admission, a new left-sided weakness was noted. A repeat CT brain scan showed a new hypodensity in the right frontal and parietal regions with mild effacement in keeping with an acute right middle cerebral artery territory infarct. Secondary stroke prophylaxis treatment was initiated as well as rehabilitation by physiotherapy and occupational therapy teams. On day 11 of admission and treatment, the patient demised. Cause of death was documented as VZV encephalitis complicated by Ramsay-Hunt syndrome and cerebral infarct on a background of advanced immunosuppression.

**TABLE 2:** Cerebrospinal fluid results of the three cases.

Case	Protein^†^ (g/L)	Lymphocytes (µL)	Polymorphs (µL)	Varicella Zoster PCR
1	0.37	1	0	Positive
2	1.01	499	0	Positive
3	2.97	431	217	Positive

PCR, polymerase chain reaction.

† Normal reference range = 0.15–0.4.

## Discussion

This case series highlights a few different neurological manifestations of VZV reactivation. It describes the clinical presentation, investigations, clinical course, treatment and outcomes of three selected cases, which presented to New Somerset Hospital in Cape Town, South Africa, between January 2022 and September 2023. Varicella zoster virus can affect the central nervous system (CNS) in a variety of ways. The most common neurological manifestation of VZV reactivation is severe inflammation of the brain parenchyma, known as encephalitis. Other manifestations include cerebellar ataxia secondary to post-infectious meningoencephalitis, vasculitis, myelitis and rarely Guillain-Barre syndrome.^8^ The three cases described in this case series present unique manifestations of VZV reactivation. The majority of literature describes the presentations of encephalitis and meningitis. Encephalitis and meningitis have a higher incidence in adults, whereas in children the most common CNS complication is cerebellitis.^9^ A well-recognised post infective complication of herpes zoster is post-herpetic neuralgia, which is commonly treatment refractory.^8^ According to a World Health Organization 2022 report, encephalitis occurs in about 1 in every 33 000–505 000 cases of varicella infection.^10^ Varicella zoster virus encephalitis mortality rate is higher in immunosuppressed patients compared to immunocompetent patients.^11,12^ In congruence with the available literature, this was also found to be the case with our patient described in case 3.

The main two factors for varicella reactivation is advanced age and immunosuppression with its relation to declined T-cell-mediated immunity.^4^ Individuals who were subject to tissue transplants are also at an increased risk with an incidence rate of 17:1000.^13^

A commonality among the three cases is that each patient received a short course of prednisone prior to worsening of their conditions. A causal link has been attributed to the development of disseminated varicella zoster disease in immunocompetent and immunosuppressive adults and children who have been treated early with low to high doses of corticosteroids for other medical conditions, whom later had been diagnosed with varicella reactivation, as concluded by published population-based studies.^14,15^ It is recognised that corticosteroid treatment causes lymphopenia with an associated reduction in circulating T-cells as well as a reduction in monocyte counts. This effect on innate and adaptive immunity is believed to increase the likelihood and severity of varicella zoster infection.^15^ Clinical data, however, interestingly show that the use of adjunctive corticosteroids with intravenous acyclovir in the treatment of VZV reactivation results in more favourable neurological outcomes.^16,17,18^

A widely common clinical feature of the various neurological manifestations of VZV is the presence of a vesicular rash. In the above adult cases described, all the patients had the presence of an active painful vesicular pruritic rash isolated to a specific dermatomal region. Two of the cases described include individuals who had advanced HIV disease with markedly low CD4 counts. Varicella zoster virus is a T-cell immunity-related disease. Human immunodeficiency virus kills CD4 cells, also known as CD4 T helper cells. Immune dysregulation and abnormal peripheral T lymphocyte subsets have been found in patients with acute VZV infection.^19^

The one patient described above who tested negative for HIV had complete and swift resolution of disease after 6 days of intravenous acyclovir, out of a total 10-day treatment duration. On the other hand, both immunosuppressed patients described above had longer and more complex treatment durations and neither achieved full resolution of the disease. Literature has described increased mortality related to immunocompromised individuals who have varicella infection, which has been coherent to the evidence shown in this case series.

Neurological manifestations and complications of VZV reactivation described in the clinical cases above include transverse myelitis, vasculitis and stroke, cranial nerve pathology and acute psychosis. Transverse myelitis is a rare neurological manifestation of VZV with an incidence of 0.3%.^20^ Other viruses that can cause transverse myelitis include Herpes Simplex 1 and 2, Cytomegalovirus, Enterovirus and Epstein-Barr virus. Transverse myelitis is a rapidly progressing condition, which occurs secondary to an infective or inflammatory response.^21^ Transverse myelitis can be because of infective and non-infective causes. Non-infective causes include connective tissue diseases, multiple sclerosis and vitamin B12 deficiency. Theories regarding immunopathology of infection-related transverse myelitis include direct microbial invasion and injury to spinal cord, immune-mediated injury to spinal cord secondary to direct infection and systemic infection with secondary systemic inflammatory reaction causing damage to neural tissue.^22^ This pathology results in motor weakness, sensory fall-out and autonomic dysfunction.^22^ There is evidence to suggest an association between immune status and transverse myelitis prognosis. Transverse myelitis secondary to VZV infection was found to be more common in immunocompromised patients with poorer outcomes compared to immunocompetent patients.^23^

The second case portrays an example of infection induced encephalopathy with the presentation of first-onset psychosis. Pathogenesis theories described in previous publications include dysfunction in cerebral blood flow, neurotransmission and energy metabolism. Release of systemic inflammatory cytokines, disruption in blood–brain barrier integrity and endothelial dysfunction have been described to cause microvascular dysfunction and microglial activation.^24^ Microvascular dysfunction has been shown to impair cerebral blood flow in frontal and temporal lobes as seen by studies focussing on imaging findings related to septic encephalopathy.^25^ Microglial activation is believed to impair neurotransmitter metabolism.^26^ More recent studies have shown an association with limbic encephalitis, CSF lymphocytic pleocytosis and subacute neuropsychiatric symptoms.^27^

Varicella zoster virus infection has been reported to commonly affect the distribution of the facial nerve, trigeminal nerve and vestibulocochlear nerve. The description of Ramsay-Hunt syndrome in the third clinical case describes this presentation.^28^ Vestibulocochlear neuropathy features can be present, which includes tinnitus, sensorineural hearing loss and vertigo. This commonly occurs in the presence of facial paralysis. Ophthalmic complications can occur if the ophthalmic branch of the trigeminal nerve is affected. These include optic neuritis, glaucoma, keratitis, scleritis, iritis and retinitis.^2,28^ A further neurological complication of VZV was evident in this clinical case in the form of ischaemic stroke. Acquired antibody-mediated coagulation disorders have been postulated in reports of VZV infection with risk of thromboembolism.^29^ Additionally multiple reports have documented vasculopathies as a complication of VZV.^30^

As demonstrated, VZV infection carries the possibility of severe neurological consequences. A vaccination programme may serve to limit the many complications. Vaccination aims to decrease the incidence and morbidity of varicella zoster CNS infection. Although there is use of the vaccination in other regions of the world, it is currently not part of the Expanded Programme of Immunization in South Africa, according to the National Institute for Communicable Diseases. The vaccination can, however, be given to vulnerable populations post exposure.

## Conclusion

Varicella zoster infection can prove fatal if not managed correctly. Infected immunocompromised individuals have been shown to have poorer prognoses. The presentation of vesicular pruritic rash with altered mental state, vomiting, severe headache, new-onset seizures and/or focal neurology should alert clinicians to consider VZV reactivation. Attention has been drawn to specific diagnostic measures, which include early lumbar puncture with CSF microscopy and VZV PCR testing, as well as specialised brain imaging. Favourable outcomes have been proven with the treatment of acyclovir IV, with extended courses in immunocompromised patients. Adjunctive steroid treatment proves useful in selected neurological zoster cases, while caution has been advised with early prescription of steroids for other medical conditions because of its link to development of severe varicella disease in individuals with reactivation.
